# Duration and clinical outcome of dual antiplatelet therapy after percutaneous coronary intervention: a retrospective cohort study using a medical information database from Japanese hospitals

**DOI:** 10.1007/s12928-021-00833-z

**Published:** 2022-02-09

**Authors:** Hiroyoshi Yokoi, Eisei Oda, Kazuki Kaneko, Kenta Matsubayashi

**Affiliations:** 1 Cardiovascular Medicine Center, Fukuoka Sanno Hospital, 3-6-45 Momochihama, Sawara-ku, Fukuoka, 814-0001 Japan; 2 International University of Health and Welfare, Tochigi, Japan; 3 StatLink Medical Statistics Consulting Service, Tokyo, Japan; 4 EBM Unit, Medical Data Vision Co., Ltd., Tokyo, Japan; 5 Clinical Development Department II, Daiichi Sankyo Co., Ltd., Tokyo, Japan

**Keywords:** Retrospective cohort study, Medical database, Percutaneous coronary intervention, Dual antiplatelet therapy, Japan

## Abstract

In this real-world, retrospective cohort study of 9753 patients in Japan prescribed dual antiplatelet therapy (DAPT) following percutaneous coronary intervention (PCI), we investigated DAPT duration and determined factors associated with early DAPT discontinuation and with event rates in patients who discontinued DAPT. The study period was April 1, 2012–March 31, 2018; endpoints comprised composite efficacy [death, myocardial infarction (MI), and stroke] and bleeding (intracranial, gastrointestinal, and requiring transfusion) endpoints. Overall, 68.8% of patients were continuing DAPT at 3 months post-PCI. Patients without major efficacy or safety events within 3 months after index PCI were included in a landmark analysis set (LAS; *n* = 7056), and categorized as DAPT ≥ 3 months (continuation) versus < 3 months (discontinuation). In the two LAS analysis groups, there was no difference in the composite bleeding endpoint (*P* = 0.067), although the incidence of the composite efficacy endpoint was higher in the discontinuation group (*P* < 0.001). In multivariate regression analysis, age ≥ 75 years, minor bleeding after PCI, history of cerebral infarction, history of cerebral or gastrointestinal bleeding, atrial fibrillation, dialysis, and anticoagulant use after PCI were associated with early DAPT discontinuation. Acute coronary syndrome, history of MI, kidney disorder, and anticoagulant use after PCI were associated with the composite efficacy endpoint in the discontinuation group. In conclusion, early DAPT discontinuation is more likely in patients at high bleeding risk, but may influence the occurrence of ischemic events in these patients. Determination of DAPT duration should take into account potential ischemic risk, even in patients at high bleeding risk.

**Supplementary Information:**

The online version contains supplementary material available at 10.1007/s12928-021-00833-z.

## Introduction

Coronary artery disease (CAD) is the second leading cause of death in Japan (15.2%), and the incidence of ischemic heart disease (IHD) in Japan is increasing, with more than 200,000 deaths due to heart disease (excluding hypertension) reported in 2017 [1]. Percutaneous coronary intervention (PCI) is the primary treatment for IHD [2], and dual antiplatelet therapy (DAPT) is an integral part of medical treatment post-PCI for the prevention of reinfarction [3, 4].

American Heart Association (AHA) guidelines recommend DAPT for varying durations depending on whether the patient has stable IHD or acute coronary syndrome (ACS) [4]. According to Japanese guidelines, the duration of DAPT should be determined based on individual patient risks, such as bleeding risk or ischemic risk; however, in general, the recommended duration of DAPT is < 3 months [5]. Many studies have been conducted to determine the optimal duration of DAPT by comparing efficacy and safety between short- and long-term DAPT, but the results reported vary considerably depending on the study design, methodology, and patient population [6].

Until recently, few studies have considered real-world clinical settings, resulting in challenging clinical decision-making to determine which patients are eligible for DAPT treatment [7, 8]. The current focus on DAPT-scoring systems may eventually assist in this process. Still, in the short term, the proliferation in competing systems for risk definition and inconsistent outcomes has added a further complexity level [5, 9–12]. Considering that the risk of bleeding is higher and the risk of ischemic events is lower in Asians than in non-Asians [13, 14], the balance between ischemic and bleeding events in Japan appears to be different from that in other countries. Therefore, it would be beneficial for physicians to understand the findings from the real-world setting in Japan to apply them in clinical practice. Hence, there is a need to clearly determine the background characteristics of PCI patients and the actual use and prognosis of DAPT after PCI in the Japanese clinical practice setting.

The objectives of this retrospective, observational study were to investigate the actual duration of DAPT in PCI patients in the real-world setting and to determine patient background factors leading to early discontinuation of DAPT (within 3 months of initiation). In addition, the incidences of bleeding events and ischemic events were compared between patients who discontinued DAPT and those who continued treatment, and we explored potential risk factors for ischemic events in patients who discontinued DAPT.

## Methods

### Study design

This was a retrospective, observational cohort study conducted in Japan to evaluate patients with CAD who were prescribed DAPT (aspirin and thienopyridine) following PCI. The study period for data extraction from the medical information database was from April 1, 2012 to March 31, 2018. The follow-up period was ≥ 91 days, and there was no upper limit.

### Data sources

All data (background, treatment, and follow-up) used in this analysis were obtained from a hospital-based composite database containing administrative and laboratory data stored in hospital electronic information systems (Medical Data Vision Co., Ltd., Tokyo, Japan). This database contains anonymized patient data on both inpatient and outpatient health insurance claims for over 18 million patients in 338 hospitals throughout Japan (as of March 2018). The target for this study was acute-phase hospitals (i.e., those eligible for Diagnosis Procedure Combination; DPC) throughout Japan, and the 338 DPC hospitals included in the database account for approximately 20% of all DPC hospitals across Japan.

The information in this database is obtained from hospitals with permission for secondary use and provides real-world disease treatment and prescription data. A key characteristic of the data source is that collection of data on acute diseases and elderly people is a simple process compared with other databases (e.g., insurance claims). In addition, the data are validated for errors such as sex and age when they are provided from hospitals. As the database covers acute medical institutions throughout Japan without any bias relating to geographical location, selection bias is minimized. This study focused on healthcare data for PCI-treated patients who visited medical institutions for acute care.

### Patients

Eligible patients were those whose information was included in the database during the study period and who met the following inclusion criteria: a record of a definitive diagnosis of CAD, a record of PCI treatment date, and a record of starting DAPT on the day of or the day following PCI. The first PCI accompanied by the start of DAPT was defined as the index PCI, and the date of the index PCI was defined as the index date (ID). Eligible patients were also required to have had an initial hospital visit within 30 days prior to the ID (to allow the collection and analysis of background factors, such as medical complications and comorbidities), at least one hospital visit in the 90 days before the ID, a stent placed during index PCI, and a prescription record of an adenosine diphosphate inhibitor at least once after the discharge date. The minimum duration of DAPT was not needed for inclusion. Patients with no available information prior to the ID were not eligible for inclusion, but there were no other formal exclusion criteria. Each patient’s follow-up period commenced on the ID and ended on the last hospital visit or last discharge date on or before June 30, 2017, or for inpatients on March 31, 2018.

This study used de-identified data retrieved in an unlinked manner from the Medical Data Vision automated hospital information database. Therefore, the study was exempt from obtaining informed consent from individual patients according to the local ethical guidelines for epidemiological research [15].

### Variables and endpoints

Data were collected on patient background (sex, age, medical history, and history of revascularization treatment), PCI information, and medications. The main variable investigated was the duration of DAPT, as recorded in the database. DAPT discontinuation was determined when the prescription of DAPT was not continued for more than 30 days.

For landmark analysis, DAPT duration was categorized as follows: DAPT ≥ 3 months (continuation) versus DAPT < 3 months (discontinuation). The endpoints of the present study were a composite efficacy endpoint of death, myocardial infarction (MI), and stroke, and a composite bleeding endpoint of intracranial bleeding, gastrointestinal bleeding, and bleeding requiring transfusion. Events were adjudicated using specified sources (master/dictionary) and conditions, as shown in Supplementary Appendix 1.

### Statistical analysis

The full analysis set (FAS) comprised patients who had an elective PCI and those who had PCI for ACS. Patients included in the FAS, who did not have major efficacy events (i.e., all death, cardiovascular death, MI, stroke, ischemic stroke, and stent thrombosis) or major safety events (i.e., intracranial bleeding, gastrointestinal bleeding, and bleeding requiring transfusion) within 3 months after the index PCI, were included in the landmark analysis set (LAS); patients with a follow-up period of ≤ 90 days and those with a DAPT duration of ≤ 90 days plus no visit for 30 days after DAPT discontinuation were excluded from the LAS.

Patient background and clinical characteristics were summarized for the FAS and the LAS according to DAPT continuation/discontinuation group at 3 months after index PCI. Multivariate logistic analyses were conducted to determine the variables affecting DAPT continuation. The initial model incorporated 16 exploratory variables, including 15 baseline factors (such as age, history of cerebral infarction, and minor bleeding within 3 months of PCI), and a backward elimination procedure was used to select significant factors. Kaplan*–*Meier methodology was used to estimate the proportional incidences of efficacy events and bleeding events at each timepoint after index PCI, by group. Between-group differences for each event incidence were evaluated by the log-rank test. Multivariate Cox regression analysis was used to identify factors associated with the occurrence of the composite efficacy events for each group; again, the backward elimination procedure was used to select significant factors from the initial model.

All statistical analyses were performed by AC Medical Inc. (Tokyo, Japan) using SAS software version 9.4 (SAS Institute Inc., Cary, NC, USA).

## Results

### Patients

**Fig. 1:**
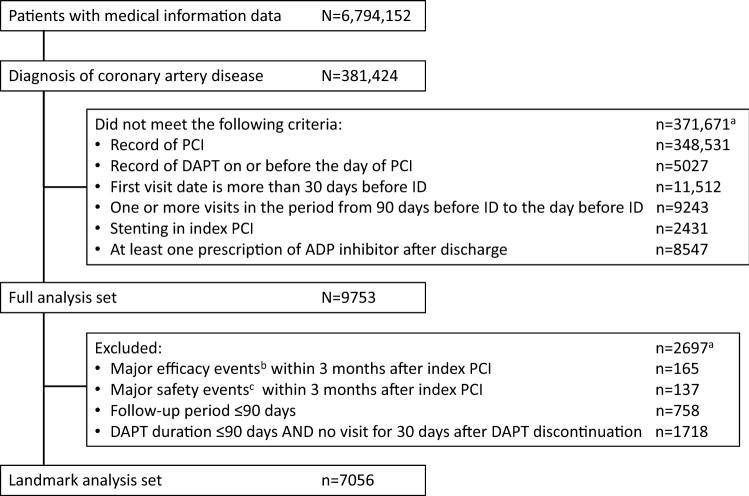
Selection of study population and analysis sets. ^a^ Patients could be excluded for more than one reason. ^b^ Efficacy events were all death, cardiovascular death, myocardial infarction, stroke, ischemic stroke, and stent thrombosis. ^c^ Safety events were intracranial bleeding, gastrointestinal bleeding, and bleeding requiring transfusion. *ADP* adenosine diphosphate, *DAPT* dual antiplatelet therapy, *ID* index date, *PCI* percutaneous coronary intervention

**Fig. 2:**
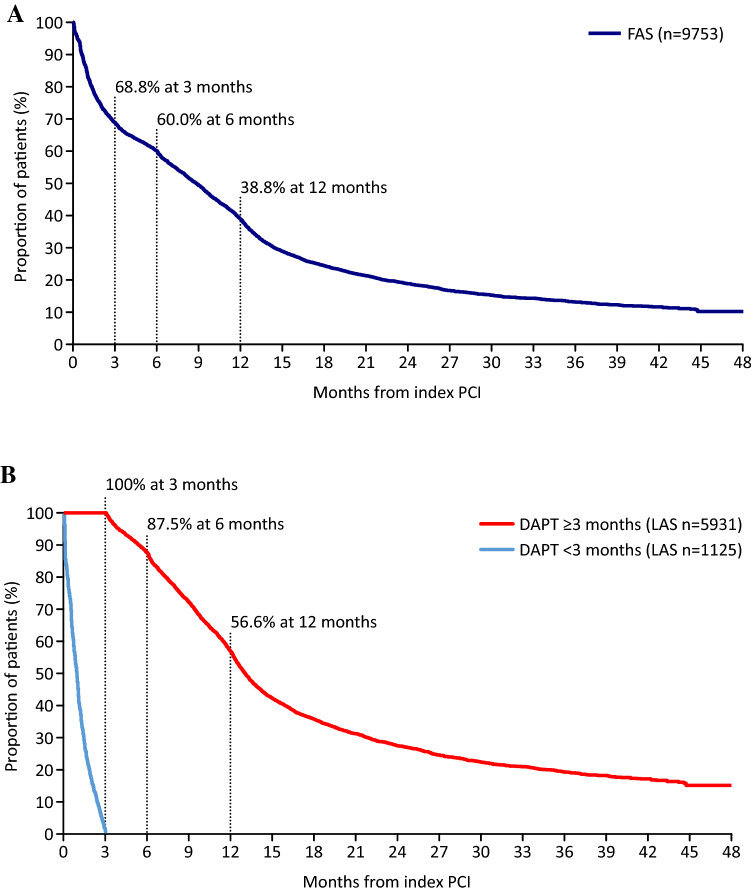
Kaplan–Meier curve of the proportion of patients on DAPT in the FAS (**A**) and in the LAS (DAPT < 3 months and ≥ 3 months) (**B**) after index PCI. *DAPT* dual antiplatelet therapy, *FAS* full analysis set, *LAS* landmark analysis set, *PCI* percutaneous coronary intervention

As shown in Fig. 1, a total of 9753 patients were included the FAS and 7056 in the LAS. The demographic and clinical characteristics of patients in each analysis set are shown in Table 1. Many characteristics were similar for patients in the FAS, the LAS DAPT discontinuation group, and the LAS DAPT continuation group; the respective mean ages in these three groups were 71.2, 72.9, and 70.6 years, and the respective proportions of females were 24.6%, 26.2%, and 23.5%. However, more patients in the LAS DAPT discontinuation group were aged ≥ 75 years (47.7%) compared with the FAS (40.2%) and the LAS DAPT continuation group (37.1%). Most patients had undergone elective PCI (FAS, 84.4%; LAS DAPT discontinuation group, 81.9%; LAS DAPT continuation group, 84.1%). In terms of stent type, drug-eluting stent was most common (FAS, 95.4%; LAS DAPT discontinuation group, 91.0%; LAS DAPT continuation group, 96.0%) and most patients had a single stent (69.2% in all three groups). In the FAS, the proportion of patients who continued DAPT after the index PCI was 68.8% at 3 months (Fig. 2A). Adenosine diphosphate receptor inhibitors used after the index PCI in the FAS were clopidogrel (including clopidogrel/aspirin combination tablet) 62.7%, prasugrel (3.75 mg or 2.5 mg) 34.9%, and other 2.4%; no patients (0%) used ticagrelor. The proportion of patients on DAPT in the LAS (DAPT < 3 months and ≥ 3 months) after index PCI is shown in Fig. 2B. In the group with DAPT ≥ 3 months, 56.6% continued DAPT at 12 months.

**Table 1:** Demographic and clinical characteristics of the FAS and the LAS

Characteristic	FAS (n = 9753)	LAS (n = 7056)	
		DAPT < 3 months (n = 1125)	DAPT ≥ 3 months (n = 5931)
Sex, female, *n* (%)	2404 (24.6)	295 (26.2)	1393 (23.5)
Age, years, mean ± SD	71.2 ± 10.0	72.9 ± 9.6	70.6 ± 10.0
≥ 75 years, *n* (%)	3916 (40.2)	537 (47.7)	2201 (37.1)
Prior revascularization, *n* (%)			
No	6936 (71.1)	760 (67.6)	4432 (74.7)
Yes	338 (3.5)	73 (6.5)	159 (2.7)
Unknown	2479 (25.4)	292 (26.0)	1340 (22.6)
PCI classification, *n* (%)			
Acute MI	593 (6.1)	74 (6.6)	384 (6.5)
Unstable angina	931 (9.5)	128 (11.4)	557 (9.4)
Elective PCI	8227 (84.4)	921 (81.9)	4990 (84.1)
Stent			
BMS	422 (4.3)	98 (8.7)	218 (3.7)
DES	9300 (95.4)	1024 (91.0)	5693 (96.0)
BMS + DES	31 (0.3)	3 (0.3)	20 (0.3)
Number of stents			
Single	6746 (69.2)	779 (69.2)	4106 (69.2)
Multiple	3007 (30.8)	346 (30.8)	1825 (30.8)
Medical history^a^			
Hypertension	8219 (84.3)	925 (82.2)	5047 (85.1)
Diabetes mellitus	3179 (32.6)	357 (31.7)	2084 (35.1)
Dyslipidemia	7985 (81.9)	854 (75.9)	5078 (85.6)
Heart failure	1074 (11.0)	142 (12.6)	583 (9.8)
Atrial fibrillation	1472 (15.1)	248 (22.0)	828 (14.0)
Stroke	377 (3.9)	60 (5.3)	194 (3.3)
Cerebral hemorrhage	50 (0.5)	5 (0.4)	22 (0.4)
Gastrointestinal bleeding	135 (1.4)	35 (3.1)	62 (1.0)
Gastric ulcer	684 (7.0)	98 (8.7)	419 (7.1)
Peripheral artery disease	413 (4.2)	73 (6.5)	236 (4.0)
Liver disorder	1195 (12.3)	124 (11.0)	826 (13.9)
Kidney disorder	1866 (19.1)	289 (25.7)	1043 (17.6)
Dialysis	601 (6.2)	138 (12.3)	267 (4.5)
Medical treatment^a^			
Anticoagulant after PCI			
Warfarin	618 (6.3)	98 (8.7)	366 (6.2)
NOAC	803 (8.2)	150 (13.3)	433 (7.3)
Proton pump inhibitor	5982 (61.3)	628 (55.8)	3844 (64.8)

^a^Some patients fall into more than one category

*BMS* bare-metal stent, *DAPT* dual antiplatelet therapy, *DES* drug-eluting stent, *FAS* full analysis set, *LAS* landmark analysis set, *MI* myocardial infarction, *NOAC* non-vitamin K oral anticoagulant, *PCI* percutaneous coronary intervention, *SD* standard deviation

### Clinical events

The multivariate logistic analysis of factors associated with DAPT discontinuation at 3 months after index PCI is shown in Table 2. Factors associated with early DAPT discontinuation were age ≥ 75 years; minor bleeding after PCI; history of cerebral infarction; history of cerebral or gastrointestinal bleeding; atrial fibrillation; dialysis; and use of anticoagulants after PCI. Factors whose absence was associated with early DAPT discontinuation were hypertension; dyslipidemia; liver disorder; and use of proton pump inhibitors.

**Table 2:** Multivariate logistic regression analyses (backwards elimination) of factors associated with DAPT discontinuation at 3 months after index PCI (LAS; *N* = 7056)

Factor	Category	N	Patients who continued DAPT at 3 months after index PCI		Odds ratio		
			*n*	% (95% CI)	Point estimate	(95% CI)	*P*-value
LAS		7056	5931	84.1 (83.2, 84.9)			
Age	< 75 years	4318	3730	86.4 (85.3, 87.4)	Reference		
	≥ 75 years	2738	2201	80.4 (78.8, 81.9)	0.653	(0.572, 0.746)	< 0.001
Minor bleeding^a^ after PCI	No	7008	5904	84.2 (83.4, 85.1)	Reference		
	Yes	48	27	56.3 (41.2, 70.5)	0.307	(0.167, 0.563)	< 0.001
History of cerebral infarction	No	6802	5737	84.3 (83.5, 85.2)	Reference		
	Yes	254	194	76.4 (70.7, 81.5)	0.607	(0.446, 0.825)	0.001
History of cerebral or gastrointestinal hemorrhage	No	6933	5847	84.3 (83.5, 85.2)	Reference		
	Yes	123	84	68.3 (59.3, 76.4)	0.456	(0.304, 0.685)	< 0.001
Hypertension	No	1084	884	81.5 (79.1, 83.8)	Reference		
	Yes	5972	5047	84.5 (83.6, 85.4)	1.309	(1.098, 1.561)	0.003
Dyslipidemia	No	1124	853	75.9 (73.3, 78.4)	Reference		
	Yes	5932	5078	85.6 (84.7, 86.5)	1.492	(1.268, 1.755)	< 0.001
Atrial fibrillation	No	5980	5103	85.3 (84.4, 86.2)	Reference		
	Yes	1076	828	77.0 (74.3, 79.4)	0.798	(0.643, 0.991)	0.041
Liver disorder	No	6106	5105	83.6 (82.7, 84.5)	Reference		
	Yes	950	826	86.9 (84.6, 89.0)	1.328	(1.080, 1.631)	0.007
Dialysis	No	6651	5664	85.2 (84.3, 86.0)	Reference		
	Yes	405	267	65.9 (61.1, 70.5)	0.321	(0.256, 0.403)	< 0.001
Use of anticoagulant (warfarin or NOAC) after PCI	No	6041	5159	85.4 (84.5, 86.3)	Reference		
	Yes	1015	772	76.1 (73.3, 78.7)	0.642	(0.516, 0.800)	< 0.001
Use of PPI	No	2584	2087	80.8 (79.2, 82.3)	Reference		
	Yes	4472	3844	86.0 (84.9, 87.0)	1.572	(1.376, 1.797)	< 0.001

The table shows factors with significance only. Additional factors that were evaluated and found not to be significant were PCI classification, number of stents, history of myocardial infarction, diabetes mellitus, and kidney disorder

^a^Minor bleeding was defined as any bleeding event other than a major safety event (where major safety events were intracranial bleeding, gastrointestinal bleeding, and bleeding requiring transfusion)

*CI* confidence interval, *DAPT* dual antiplatelet therapy, *LAS* landmark analysis set, *NOAC* non-vitamin K oral anticoagulant, *PCI* percutaneous coronary intervention, *PPI* proton pump inhibitor

**Fig. 3:**
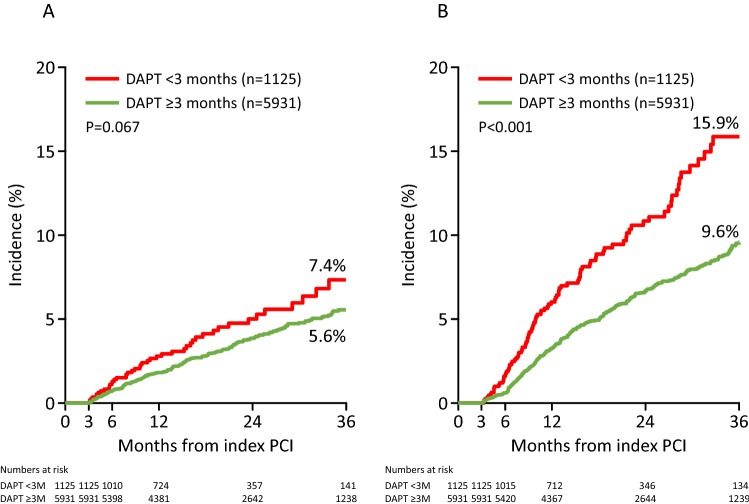
Incidences of composite endpoints in the LAS. **A** Composite bleeding endpoint (intracranial bleeding, gastrointestinal bleeding, and bleeding requiring transfusion); **B** composite efficacy endpoint (death, myocardial infarction, and stroke). *DAPT* dual antiplatelet therapy, *LAS* landmark analysis set, *M* months, *PCI* percutaneous coronary intervention

The incidences of the composite bleeding and efficacy endpoints in the LAS are shown in Fig. 3A,B. Up to 36 months after the index PCI, there were no significant differences in the composite bleeding endpoint (intracranial bleeding, gastrointestinal bleeding, and bleeding requiring transfusion) between the DAPT discontinuation group and the DAPT continuation group. However, a significant between-group difference was observed in the incidence of the composite efficacy endpoint (death, MI, and stroke); at 36 months after the index PCI, the incidence was 15.9% in the LAS DAPT discontinuation group and 9.6% in the LAS DAPT continuation group (*P* < 0.001).

A multivariate Cox regression analysis of factors associated with composite efficacy events is shown in Table 3. In patients in the LAS receiving DAPT for < 3 months, factors associated with the composite efficacy endpoint of death, MI, and stroke were ACS; history of MI; kidney disorder; and use of anticoagulants after PCI.

**Table 3:** Multivariate Cox regression analyses (backwards elimination) of factors associated with the composite efficacy endpoint of death, myocardial infarction, and stroke in patients receiving DAPT < 3 months in LAS (*N* = 1125)

Factor	Category	*N*	Event, *n* (%)	Hazard ratio		
				Point estimate	95% CI	*P*-value
PCI classification^a^	ACS	204	29 (14.2)	Reference		
	non-ACS	921	73 (7.9)	0.486	(0.314, 0.752)	0.001
History of myocardial infarction	No	1015	78 (7.7)	Reference		
	Yes	110	24 (21.8)	2.964	(1.866, 4.709)	< 0.001
Kidney disorder	No	836	62 (7.4)	Reference		
	Yes	289	40 (13.8)	2.110	(1.409, 3.161)	< 0.001
Use of anticoagulants (warfarin or NOAC) after PCI	No	882	71 (8.0)	Reference		
	Yes	243	31 (12.8)	1.624	(1.059, 2.491)	0.026

^a^ACS includes acute myocardial infarction or unstable angina

The table shows factors with significance only. Additional factors that were evaluated and found not to be significant were age, minor bleeding after PCI (defined as any bleeding event other than a major safety event), number of stents, history of cerebral infarction, history of cerebral or gastrointestinal hemorrhage, hypertension, diabetes mellitus, dyslipidemia, atrial fibrillation, liver disorder, dialysis, and use of PPI

*ACS* acute coronary syndrome, *CI* confidence interval, *DAPT* dual antiplatelet therapy, *LAS* landmark analysis set, *NOAC* non-vitamin K oral anticoagulant, *PCI* percutaneous coronary intervention, *PPI* proton pump inhibitor

## Discussion

This was a retrospective cohort study to examine the real-world duration and use of antiplatelet treatment in PCI patients in Japan. Our findings indicate that the proportion of patients who discontinued DAPT within 3 months after index PCI was about 30%, although treatment guidelines at that time recommended a 12-month duration of DAPT after implantation of a drug-eluting stent [16–19].

In the present study, an analysis of factors associated with early DAPT discontinuation found that there was a notable overlap with high bleeding risk factors in PCI patients [as per the Japanese version of high bleeding risk assessment criteria featured in the Japanese Circulation Society (JCS) 2020 guideline [5], which was developed based on the Academic Research Consortium for High Bleeding Risk], including older age, cerebral infarction or bleeding, use of anticoagulants, and dialysis. We conducted a landmark analysis, excluding patients who had major bleeding within 3 months of the index PCI, and found that DAPT durations remained short (< 3 months) in patients with high bleeding risk even without recent major bleeding.

Although the Japanese guidelines at the time when the present study was conducted recommended that the duration of DAPT after PCI should be 12 months [16–19], our analysis revealed that almost one-third of real-world patients actually received DAPT for < 3 months. This may have been a result of the publication of revised AHA guidelines [4] during the preset study period, in which the duration of DAPT after PCI was modified from 12 down to 6–12 months. The revised AHA guidelines also recommended that, for patients at a high risk of bleeding (e.g., stable IHD), DAPT duration could be further shortened to a duration of 3 months [4]. Thus, although the present study was conducted before the revision of the Japanese guidelines, it is possible that Japanese physicians were already decreasing DAPT duration for their patients in line with the global trend.

We estimated the incidence of clinical events in the groups of patients who continued and discontinued DAPT and found no significant difference between the groups for the composite bleeding endpoint. Since the patients at high risk of bleeding were most likely to discontinue DAPT early, we can infer that shortening DAPT duration prevents bleeding events in this patient population. Conversely, however, we observed an increased incidence of ischemic events in the patients who discontinued DAPT within 3 months of the index PCI. We found a clear overlap with previously reported risk factors for thrombosis when we investigated factors associated with ischemic events in patients with early DAPT discontinuation. ACS and chronic kidney disease are specified as a risk factor for coronary thrombotic events by the JCS 2020 guideline [5]. A history of MI, one of the factors associated with ischemic events in the present study, may indicate a history of PCI or coronary artery bypass graft, which are also considered risk factors for thrombosis by the JCS [5]. Furthermore, the use of anticoagulants may imply the existence of atrial fibrillation, which is another ischemic risk factor in patients undergoing PCI [20, 21]. This result suggests that in patients with high bleeding risk, DAPT duration < 3 months may be insufficient to prevent ischemic events, particularly in patients at high ischemic risk. Rather than simply deciding on a DAPT duration of 1–3 months because of high bleeding risk, the DAPT duration should be decided based on each patient’s ischemic and bleeding risks, and it may be an option to continue DAPT for more than 3 months in high bleeding risk patients.

Current Japanese guidelines recommend that in patients at high bleeding risk, who are not receiving oral anticoagulants, the duration of DAPT should be limited to 1–3 months; in patients at high risk of both bleeding and thrombotic events, short-term DAPT treatment followed by P2Y_12_ receptor inhibitor monotherapy should be considered [5]. The results from SMART-CHOICE support this regimen, showing that P2Y_12_ inhibitor monotherapy after 3 months of DAPT resulted in noninferior rates of major adverse cardiac and cerebrovascular events compared with prolonged DAPT [22]. In another study that analyzed 3045 Japanese patients undergoing PCI, DAPT followed by clopidogrel monotherapy resulted in a significantly lower incidence of bleeding events without increasing cardiovascular events compared with continuous DAPT [23]. Moreover, in a recent study comparing prasugrel single antiplatelet therapy following DAPT versus continuous DAPT in Japanese PCI patients with high bleeding risk, prasugrel was shown to significantly reduce bleeding events without increasing cardiovascular events [24]. The present study results showed that the incidence of ischemic events increased in patients who discontinued DAPT early, suggesting that these patients are at high risk for both bleeding and ischemic events. For this patient population, P2Y_12_ inhibitor monotherapy may be a useful therapeutic option.

## Limitations and strengths

This study has some limitations that should be considered when interpreting the results, including possible bias inherent to the retrospective cohort design and limited generalization to other populations due to the use of data from Japanese hospitals only. As the present study was conducted before the current JCS 2020 guideline was updated (i.e., 2012–2018), the 3-month DAPT duration, which is recommended in the current guideline, may not be reflected. However, we analyzed data using a cutoff DAPT duration of 3 months; thus, the findings are applicable to the current clinical practice. In addition, the accuracy of the event definitions we used is not yet confirmed by validation research and remains to be determined in future analyses. Moreover, the database does not include death or other events that occurred at home or in other hospitals after discharge, meaning that the risk of events may be underestimated. This is a general shortcoming of medical information database research. It is also possible that the higher clinical event rates among patients who did not continue DAPT at 3 months may be the consequence of a selection bias towards individuals who were forced to prematurely cease DAPT treatment due to clinical or other events (such as complications or surgery). In addition, the exclusion of patients lacking data prior to the ID may have caused more chronic-phase patients to be included in the study, compared with acute-phase patients, potentially contributing to the observed low incidence of acute MI. Since it was not feasible to evaluate and adjust for all possible variables that may have driven the DAPT duration decision in this retrospective analysis, the results should be viewed as a complement to those obtained from randomized clinical trials. Nevertheless, given the current scarcity of real-world data, we believe that the analyses reported herein are of considerable interest to clinicians initiating DAPT treatment in patients undergoing PCI. This study has several strengths that elevate the quality of the evidence produced. The source database is not biased to a specific region in Japan and contains a large sample population that reflects Japan’s actual population [25, 26]. Furthermore, a previously published study using the same database demonstrated that the data obtained were comparable with results from a similar cohort study in Japan [27]. The source database was chosen specifically because it included acute care hospitals, which is the setting in which patients undergoing PCI are most likely to be treated. Finally, the findings from the present study may be beneficial for physicians in clinical practice as this study used real-world data from Japan, where the risk balance between ischemic and bleeding events appears to be different from that in other countries.

## Conclusion

The present study results reveal that in real-world clinical practice, patients with characteristics included in the risk scoring systems for bleeding events after PCI (Japanese version of high bleeding risk assessment criteria featured in the JCS 2020 guideline) are most likely to discontinue DAPT within 3 months of PCI. However, the incidence of ischemic events was found to be high in patients with early DAPT discontinuation. Given that patients with characteristics included in the risk scoring systems for ischemic events after PCI experienced ischemic events more frequently after DAPT discontinuation, we consider that ischemic risk should be taken into consideration when determining DAPT duration, even in patients determined to be at high bleeding risk.

## Supplementary Information

Below is the link to the electronic supplementary material.

Supplementary file1 (DOCX 29 KB)
