# Supplementary material for: Duration and clinical outcome of dual antiplatelet therapy after percutaneous coronary intervention: a retrospective cohort study using a medical information database from Japanese hospitals
Source: Cardiovasc Interv Ther. 2022 Feb 9;37(3):465–74. doi: 10.1007/s12928-021-00833-z (PMC9197891; doi:10.1007/s12928-021-00833-z)
Supplement: Supplementary file 1 — Supplementary file1 (DOCX 29 KB) [file 12928_2021_833_MOESM1_ESM.docx]

**Supplementary Appendix 1.**

**Master and dictionary used in this study**

|  | **Master / dictionary** | **Version** | **Publisher** |
| --- | --- | --- | --- |
| **Disease** | Disease Master | Added data up to July 3, 2017 to the version of January 15, 2010 | Medical Fee Information Service (Health Insurance Bureau, Ministry of Health, Labour and Welfare) |
|  | International Classification of Diseases (ICD)-10 code | Version 10 | World Health Organization |
| **Drugs** | Drug Master | Added data up to August 31, 2017 to the version of January 15, 2010 | Medical Fee Information Service (Health Insurance Bureau, Ministry of Health, Labour and Welfare) |
|  | Anatomical Therapeutic Chemical Classification (European Pharmaceutical Market Research Association) | Added data up to August 9, 2017 to the version of April 1, 2012 | Japan Pharmaceutical Information Center |
| **Medical practice** | Medical Procedure Master | Added data up to July 6, 2017 to the version of January 15, 2010 | Medical Fee Information Service (Health Insurance Bureau, Ministry of Health, Labour and Welfare) |
| **Material** | Material Master | September 1, 2017 | Medical Fee Information Service (Health Insurance Bureau, Ministry of Health, Labour and Welfare) |

**Efficacy events**

The efficacy events investigated in the present study are shown below. Events 1–6 were defined as major efficacy events.

1. All-cause death: Defined as below with date of discharge taken as date of occurrence.

- When the outcome at discharge for an inpatient is death: Yes.
- Others: No.

Note: Not applicable for patients who died during outpatient treatment.

2. Cardiovascular death: Defined as below with date of discharge taken as date of occurrence.

- When all of the following conditions are satisfied: Yes.
  - Hospitalized for “disease requiring maximum use of medical resources” that is due to efficacy events 3–6.
  - Outcome at discharge is death due to the “disease requiring maximum use of medical resources”.
- Others: No.

3. Myocardial infarction: Defined as below with date of admission taken as date of occurrence.

- When all of the following conditions are satisfied: Yes.
  - Hospitalized with an injury/illness that is classified as “myocardial infarction” in the Disease Master as the “injury/illness that triggered the hospitalization” in the hospitalization that occurred after the hospitalization that includes the index date (ID).
  - During the above hospitalization, there was a medical procedure classified as “myocardial infarction” in the Medical Procedure Master or Material Master.
- Others: No.

Note: The definition of lethal and non-lethal was not determined due to the complexity of the categorization.

4. Stroke: Defined as below with date of admission taken as date of occurrence.

- When all of the following conditions are satisfied: Yes.
  - Hospitalized with an injury/illness that is categorized as “stroke” in the Disease Master as the “injury/illness that triggered hospitalization”.
  - During the above hospitalization, there was a medical procedure classified as “stroke” in the Medical Procedure Master.
- Others: No.

Note: The definition of lethal and non-lethal was not determined due to the complexity of the categorization.

5. Ischemic stroke: Defined as below with date of admission taken as date of occurrence.

- When all of the following conditions are satisfied: Yes.
  - Hospitalized with an injury/illness that is classified as “ischemic stroke” in the Disease Master as the “injury/illness that triggered hospitalization”.
  - During the above hospitalization, there was a medical procedure classified as “ischemic stroke” in the Medical Procedure Master.
- Others: No.

Note: The definition of lethal and non-lethal was not determined due to the complexity of the categorization.

6. Stent thrombosis: Defined as below with date of admission taken as date of occurrence.

- When all of the following conditions are satisfied: Yes.
  - Hospitalized for “restenosis after coronary stenting” in the Disease Master.
  - During the above hospitalization, there was a medical procedure categorized as “stent thrombosis” in the Medical Procedure Master or Material Master.
- Others: No.

## **Safety events**

The safety events investigated in the present study are shown below. Events 1–3 were defined as major safety events.

1. Intracranial bleeding: Defined as follows with date of imaging examination taken as date of occurrence.

- When all of conditions A–D below are satisfied: Yes.

Note: Condition 1 only applies to events occurring during the index percutaneous coronary intervention (PCI) hospitalization period, and does not apply to events when readmitted.

1. Medical history
   - No diseases in the following ICD-10 disease categories occurred in the month prior to index PCI.
   - Cerebrovascular diseases (I60-69). Sequelae (I690, I691, I694) are excluded.
   - Intracranial injury (S06).
2. Imaging
   - Magnetic resonance imaging or computed tomography performed after index PCI (including day of index PCI). The second and subsequent imaging examinations during the same hospitalization period are excluded.
3. Disease categories
   - Diseases in the following ICD-10 categories occurred in the same month as imaging was performed.
   - Cerebrovascular diseases (I60-69). Sequelae (I690, I691, I694) are excluded.
   - Intracranial injury (S06).
4. Rehabilitation for cerebrovascular diseases, etc.
   - Either of the following is satisfied.
   - Rehabilitation for cerebrovascular diseases, etc. within 30 days of the date of imaging.
   - Death, etc. within 30 days of the date of imaging: No.

- Other: No.

2. Gastrointestinal bleeding: Defined as below with date of examination taken as date of occurrence.

- All of conditions A– C below are satisfied: Yes.

1. Diseases in the following ICD-10 categories did not occur in the month prior to index PCI.
   - Esophagus, stomach, and duodenal diseases (K20-K31).
   - Diseases without bleeding or perforation (K253, K257, K259, K263, K267, K269, K277, K279, K287, K289) are excluded.
   - Gastric bleeding, upper gastrointestinal bleeding (K92.2).
   - Other intestinal diseases (K55-K63).
   - Peritoneal diseases (K65-K67).
   - Melena (K92.1).
2. Any of the following examinations were performed from the ID onwards (including day of ID).
   - Gastro-duodenal fiberscopy.
   - Colon fiberscopy.
   - Fecal occult blood test.

Note: Second and subsequent examinations during same hospitalization were excluded.

1. Diseases in the following ICD-10 categories occurred in the month of performing the above examinations.
   - Esophageal, gastric, and duodenal diseases (K20-K31).
   - Diseases without bleeding or perforation (K253, K257, K259, K263, K267, K269, K277, K279, K287, K289) are excluded.
   - Gastric bleeding, upper gastrointestinal bleeding (K92.2).
   - Other intestinal diseases (K55-K63).
   - Peritoneal diseases (K65-K67).
   - Melena (K92.1).

- Others: No.

3. Bleeding requiring transfusion: Defined as below with date of transfusion taken as date of event.

- All of conditions A–D below satisfied: Yes.

1. No transfusion history in 30 days prior to index PCI (irrespective of bleeding volume).
2. Transfusion volume after index PCI (including day of index PCI) exceeding 800 mL.
3. No surgical procedure other than endoscopic hemostasis performed on day prior to or day of transfusion.
4. Excluding transfusion on day of or day following surgery.

- Others: No.
